# Some Aspects of Shear Behavior of Soft Soil–Concrete Interfaces and Its Consequences in Pile Shaft Friction Modeling

**DOI:** 10.3390/ma14102578

**Published:** 2021-05-15

**Authors:** Jakub Konkol, Kamila Mikina

**Affiliations:** Department of Geotechnics, Geology and Marine Civil Engineering, Faculty of Civil and Environmental Engineering, Gdańsk University of Technology (GUT), 11/12 Gabriela Narutowicza Street, 80-233 Gdańsk, Poland; kamila.mikina@pg.edu.pl

**Keywords:** soil-concrete interface, soft soils, skin friction, hyperbolic model, organic soil, peat

## Abstract

This paper examines the stiffness degradation and interface failure load on soft soil–concrete interface. The friction behavior and its variability is investigated. The direct shear tests under constant normal load were used to establish parameters to hyperbolic interface model which provided a good approximation of the data from instrumented piles. Four instrumented piles were used to obtain reference soil–concrete interface behavior. It was found that the variability of the friction characteristics is the highest for organic clays and the lowest for organic silts. The intact samples exhibit lower shear strength than reconstituted ones. The adhesion varies significantly depending on interface and soil type, which can result in high scatter of the skin friction prediction. The analysis of parameters variability can be used to determine the upper and lower bound of friction behavior on the interface at constant normal load condition. The backward shearing results in decrease in shear strength up to 40% of the precedent forward phase but higher initial stiffness by a factor of between 2 and 3. Presented research provides basic shear and stiffness parameters for four soft soils (organic clay, organic silt, peat, and silty loam) and gives information about variability of interface characteristics.

## 1. Introduction

### 1.1. General Considerations

The interface shear strength is important factor in geotechnical design of embankments, retaining walls or pile foundations. The engineering guidelines for determination of soil–structure interface friction properties can be found in many guidebooks [1,2]. However, some important issues arise when designers try to incorporate these guidelines in practical application. Firstly, steel interfaces are much more widely investigated than concrete ones [3,4,5,6]. Secondly, there are very limited data for friction properties of organic soil and different materials. A variability of interface friction parameters is also not often determined. There is a very low amount of data considering interface stiffness degradation with displacement. The interface friction fatigue due to large displacement or backward shearing following forward shearing is rarely considered. The ratio between forward and backward shearing is important in pile design when the direction of the shear stress can change. All above mentioned issues are addressed in the first part of this paper. In the second part, the application of laboratory test results in prediction of pile shaft friction mobilization is presented. The knowledge of soft soil–concrete interface behavior is vivid in the determination of negative [7] or positive skin friction [8]. The proper estimations of pile shaft resistance and interface stiffness degradation are crucial for successful design. The usefulness of laboratory interface testing is presented in the comparison with pile shaft friction mobilization obtained from instrumented pile tests. The parameters determined in lab tests are used in simple hyperbolic model [9] to predict the pile shaft unit resistance mobilization.

### 1.2. Current State of Knowledge

Zhang and Zhang [10] distinguish three stages in soil-structure interfaces investigation and research: (1) before the 1960s, (2) between 1960 and the 1980s, and (3) since the 1980s. Before the 1960s, interfaces were not investigated as a unique material. Between 1960 and the 1980s, the focus was laid on macroscopic frictional stress–strain relationships of the interfaces. Since the 1980s, more complex stress paths, cyclic loadings, microscopic failure mechanisms and various interfaces types (both soils and materials) were investigated. These research results in the determination of the critical roughness [5,11] and failure modes at the interface [12] for cohesive soils. The interfaces were tested under constant normal load or under constant normal stiffness conditions [13] and with different rates, which are important factors for over-consolidated cohesive soils [14] and all non-cohesive soils [15]. The most important research in terms of soil–concrete interfaces is described below.

Potyondy [16] conducted probably the first modern interface investigation focused on skin friction and adhesion measured in direct shear box. He tested friction between concrete, steel, and wood plates and sand, sandy silt and clay. The tests were made on moist and dry samples. Brandt [17] tested silty clay–concrete interfaces in small and large direct shear box. Goh [18] presented results of shearing three types of soils on rough and smooth concrete plates. Tiwari et al. [19] summarized angle of interface friction to internal friction angle ratios for different types of soils. Chen et al. [20] presented results of red clay–concrete interface shearing in large direct shear box. Canakci et al. [21] conducted interface testing between organic soil and different types of materials. Wang et al. [22] reported results of red clay–concrete cyclic shearing with small strain range. Zhang et al. [23] tested clay–concrete interfaces shear strength due to different water contents and shear rates. Wang et al. [24] investigated clay–concrete interface behavior with different roughness of concrete plates. The friction characteristics from selected previous research are summarized in Table 1 for smooth interfaces and in Table 2 for rough interfaces.

### 1.3. Aims of the Reserch

This paper examines various concrete interface properties. The most basic are the interface friction angle and adhesion. The interface stiffness degradation with displacement for different kind of soft soils is presented. The friction characteristics (shear stress ratio and interface stiffness) during backward shearing following forward are also reported. When it is possible, the standard deviation or coefficient of variation for parameters is provided. The research takes under consideration mainly organic soils (organic clay, organic silt, and peat) due to limited information in the literature, see Table 1 and Table 2. In the second part of this paper, the skin friction mobilization obtained with hyperbolic model and laboratory parameters is compared with data registered during static loading of instrumented piles.

## 2. Soils Used in Interface Testing

### 2.1. Soils Description

The cohesive soils taken under consideration in this paper are (1) silty loam, (2) organic clay, (3) organic silt, and (4) peat. These soils are commonly occurring in the Vistula Marshlands, Northern Poland [28]. All tested soils are normally consolidated. The basic physical and strength properties of soil used in interface testing are listed in Table 3 while the short description of each soil type is provided below. Silty loam (1) is inorganic soil that consists of approximately 35% clay particles and 65% silt particles. Organic clay (2) exhibit high organic matter content (10% < LOI < 30%, where LOI = Loss on Ignition) and is made of approximately 50% clay and silt particles. It contains small peat inserts which influence the permeability properties. Organic silt (3) contains moderate organic matter content (5% < LOI < 10%). It consists of silts (above 95% of particles) with very low admixture of clay or sand. Peat (4) is high organic matter soil (LOI > 30%) with fibrous structure.

### 2.2. Soil Samples Preparation

The soil samples used in this research are intact samples and reconstituted samples. The intact cohesive soil samples were taken as a block samples and then trimmed with cutting shoe and placed directly in the direct shear box. Samples were consolidated for 24 h, and then sheared. The reconstituted samples were firstly remolded and brought into the soil slurry. Then, the slurry was put into 60 × 60 mm molds. The molds were flooded with water and then gradually loaded to a stress of 35 kPa. The samples were left to be consolidated for 3 weeks. After that time, the samples were transfer from mold to direct shear box. Next, the samples were additionally consolidated for 24 h, and then sheared.

## 3. Interfaces and Direct Shear Devices

Two different interfaces were tested on three kind of direct shear box apparatus. More specific descriptions are provided below.

### 3.1. Smooth Interface

**Figure 1:**
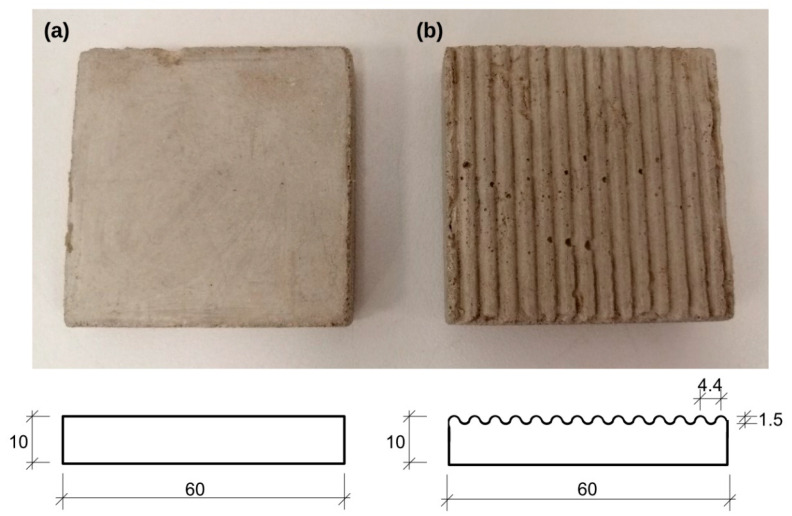
(**a**) Smooth and (**b**) rough concrete interface with idealized geometric scheme.

Smooth interface is presented in Figure 1a. It is flat, 60 × 60 mm concrete plate prepared in square metal mold. The steel plate placed at the bottom of the mold was covered with an adhesive agent. Then, the concrete mortar was poured to about half of the mold and densified. In that level the geomesh reinforcement was laid and the mold was filled with concrete to the final height of 10 mm. The concrete mortar was densified again at this point. Finished forms were secured with foil overlay and left to bind. After 7 days, the concrete plates were squeezed out of the mold and the steel plate was separated to obtain smooth interface. Then, the concrete tile was left to be completely bind within 30 days. The concrete mortar used in this study consist of cement, mineral fillers and modifiers. It reaches compressive strength of 33 MPa after 28 days. The surface roughness (R_a_) of smooth concrete interface was varying between 54 and 71 μm.

### 3.2. Rough Interface

Rough interface is presented in Figure 1b. It is serrate, 60 × 60 mm concrete plate. The preparation was analogues as in terms of smooth plate. The only difference is the application of the serrate steel plate at the bottom of the mold instead of a smooth one. The measured roughness (R_a_) of rough concrete interface was in range between 294 and 390 μm.

### 3.3. Direct Shear Box Devices

Interface shear tests were conducted in direct shear devices according to ASTM D3080/D3080M [29]. Three different types of apparatus were used. The first one was apparatus with an analogue measurement system, the second and third ones were equipped with digital measurement system. Each device consists of shear box which is divided into two rigid frames (halves) to prevent distortions during shearing. Direct shear devices are capable to provide shear force along straight plane determined by a shear box frames. The concrete interfaces were placed into the lower frame and soil samples in the upper frame. After mounting the specimens, the normal force was applied at the top of the specimen. All samples were submerged in the water during shearing and were tested according to ASTM D3080/D3080M [29] using 60 × 60 mm box and constant normal load (CNL) procedure. CNL testing is good choice for soils of low stiffness [30]. Direct shear box exhibits several disadvantages and advantages [31,32,33]. The shear plane location is fixed and shear stresses are nonuniformly distributed within specimen. However, it is advantage in interface testing of normally consolidated soft soils, where failure plane is fixed at the interface. The samples were sheared with a rate of 0.06 mm/min. This loading rate is usually achieved during static loading of the piles [34]. One can use Vermeer and Meier [35] criterion for time factor to evaluate drainage conditions during the test:

(1) $$T_{v}=\frac{kE^{\mathrm{oed}}}{\gamma_{w}l^{2}}t$$

where: T_v_ = time factor (T_v_ > 0.4 indicates drained conditions, T_v_ < 0.1 indicates undrained conditions, T_v_ between 0.1 and 0.4 suggests partially drained conditions), k = coefficient of permeability, E^oed^ = oedometric modulus, γ_w_ = unit weight of water, l = drainage length, t = loading time.

All samples were sheared under the drained conditions regarding to Equation (1) with T_v_ higher than 0.4. The above guideline was primary used. The very conservative ASTM D3080 criterion (failure time equal to 50 times t_50_, where t_50_ is time required to reach 50% of consolidation) for drained shear was not meet in every test (ASTM criterion was only meet in tests with low normal load).

### 3.4. Testing Program

The testing program is focused on several aspects: (1) the repeatability of interface shear strength, (2) the interface stiffness degradation, (3) the fatigue of interface shear strength during backward shearing following forward shearing, (4) the interface shear strength parameters and failure loads for various type of soils. The summarize of testing program is presented in Table 4.

### 3.5. Interface Shear Tests Interpretation

There are few interpretation aspects that should be considered. In this research the Mohr-Coulomb envelop is used for description of shear behavior:

(2) $$\tau_{\max}=\sigma \tan(\delta )+c_{a}$$

where: δ = angle of interface friction; τ_max_ = interface shear stress at failure; σ = normal stress applied during shearing, c_a_ = adhesion. This is the most popular approach and these kind of data were reported in Table 1 and Table 2. The variability of τ_max_ can be described by residual standard deviation S_τ_ and the variability of tan(δ) and c_a_ by the standard errors S_tan(δ)_ and S_ca_, respectively.

(3) $$S_{\tau }=\sqrt{\frac{\sum_{i=1}^{n}{\left(\tau_{i}-\tau_{\mathrm{avg}}\right)}^{2}}{n-2}}$$

(4) $$S_{\tan(\delta )}=\frac{S_{\tau }}{\sqrt{\sum_{i=1}^{n}{\left(\sigma_{i}-\sigma_{\mathrm{avg}}\right)}^{2}}}$$

(5) $$S_{\mathrm{ca}}=\sqrt{\frac{S_{\tau }{}^{2}\sum_{i=1}^{n}{\left(\sigma_{i}\right)}^{2}}{n\sum_{i=1}^{n}{\left(\sigma_{i}-\sigma_{\mathrm{avg}}\right)}^{2}}}$$

where: τ_i_ = shear stress of i-sample, τ_avg_ = average shear sample in population, n = number of samples, σ_i_ = normal stress applied during shearing of i-sample, σ_avg_ = average normal stress applied during shearing in population.

The interface tangent shear stiffness (K_i_), can be defined as tangent displacement-dependent module [22,36]:

(6) $$K_{i}=\frac{\Delta \tau }{\Delta u}$$

where: Δτ = shear stress increment; Δu = horizontal displacement increment. As the interface shear stiffness is usually stress and displacement dependent, in Table 1 and Table 2 the corresponding normal stress and displacement level are provided. The interface shear stiffness can be determined for forward and backward shearing.

## 4. Interfaces Testing Results and Discussion

### 4.1. Interface Shear Strength

**Figure 2:**
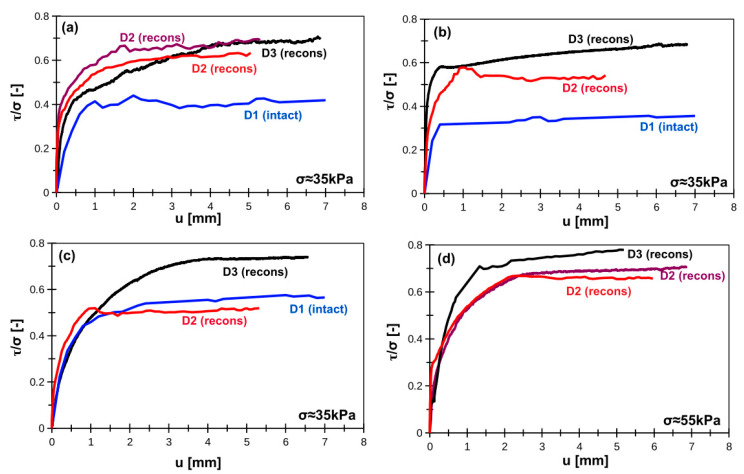
Normalized smooth concrete–soil interface shear strength for (**a**) silty loam, (**b**) organic clay, (**c**) peat, (**d**) organic silt. Note: D1, D2, D3 indicate device type; intact = intact sample, recons = reconstituted sample.

**Figure 3:**
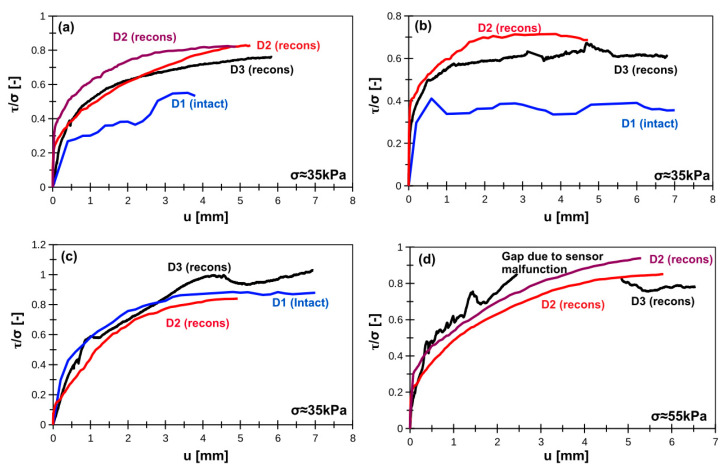
Normalized rough concrete–soil interface shear strength for (**a**) silty loam, (**b**) organic clay, (**c**) peat, (**d**) organic silt. Note: D1, D2, D3 indicate device type; intact = intact sample, recons = reconstituted sample.

**Figure 4:**
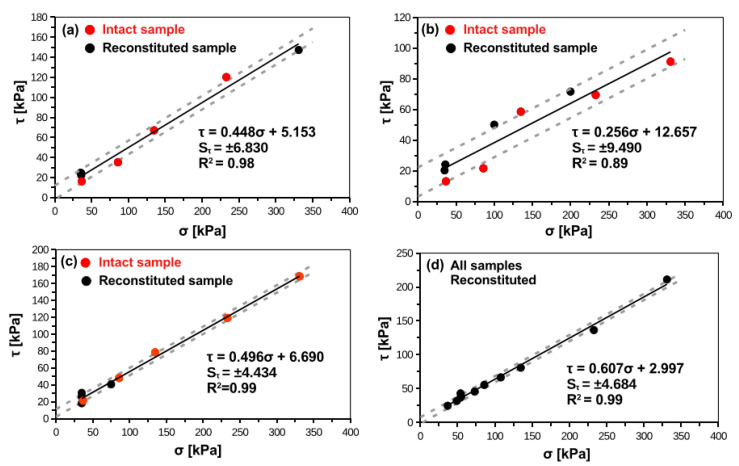
Mohr-Coulomb failure envelope of smooth concrete–soil interface for (**a**) silty loam, (**b**) organic clay, (**c**) peat, (**d**) organic silt.

The repeatability of interface shearing is presented in Figure 2 (smooth interface) and Figure 3 (rough interface). In terms of smooth interface the best reliability was achieved for organic silts. Peat, due to its fibrous and strongly anisotropic structure, can produce a relatively wide range of results. The differences in organic clay and silty loam are usually seen between intact samples and reconstituted samples. This can be related with the possible structure damage due to sampling, transportation, and storage. There is no visible influence of apparatus type on the results. In terms of rough interfaces, the reliability can be easily seen in organic silts and peats. The results for organic clay and silty loam significantly vary, usually between intact and reconstituted samples. The reason is the same as in terms of smooth interface—the differences may be induced by structure damage due to sampling, transportation and storage. The gap in D3 plot in Figure 4d (organic silt) is related to unrealistic shear stress variation probably due to stress sensor malfunction. It was one-time event during lab tests.

**Figure 5:**
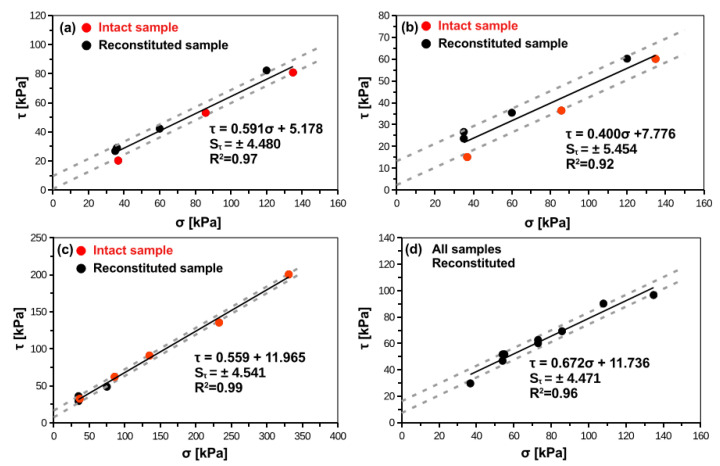
Mohr-Coulomb failure envelope of rough concrete–soil interface for (**a**) silty loam, (**b**) organic clay, (**c**) peat, (**d**) organic silt.

Figure 4 and Figure 5 present Mohr-Coulomb failure envelops for smooth and rough interface, respectively. The interface shear strength characteristics obtained from the research presented in this paper are summarized in Table 5 and Table 6. For cohesive soils, intact samples exhibit lower shear strength than reconstituted samples. For peats, there are no differences between intact and reconstituted samples. In rough interface the important adhesion part can be noticed, which is usually larger than for smooth interfaces. This aspect was seen in other studies related to the rough interface and cohesive soil, where increase in interface roughness induces increase in adhesion [20]. The obtained angles of interface friction can be characterized by low standard deviation. However, the adhesion is relatively variable parameter with coefficient of variation (COV = SD/AVG; SD = standard deviation, AVG = mean value) usually between 50% and 100%. The variability of adhesion is lower for rough interfaces than for smooth interfaces.

**Figure 6:**
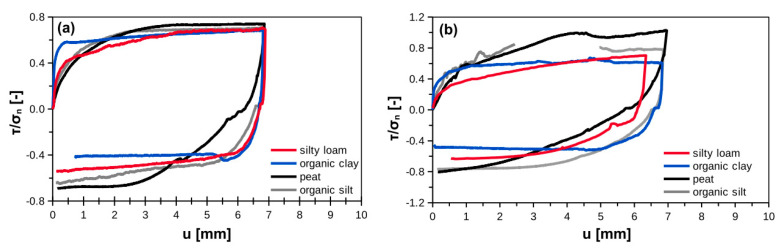
Examples of normalized shear strength during forward–backward shearing for (**a**) smooth interface and (**b**) rough interface.

Figure 6 presents the examples of forward–backward shear behavior. In all cases the behavior was quite similar. Shear strength in backward shearing was equal or lower than in forward shearing. The forward–backward decrease was the largest for organic clay and the lowest for organic silt. The relation between interface shear stresses in forward to backward shearing is similar as in reported datasets in Section 1.2.

### 4.2. Interface Stifness

**Figure 7:**
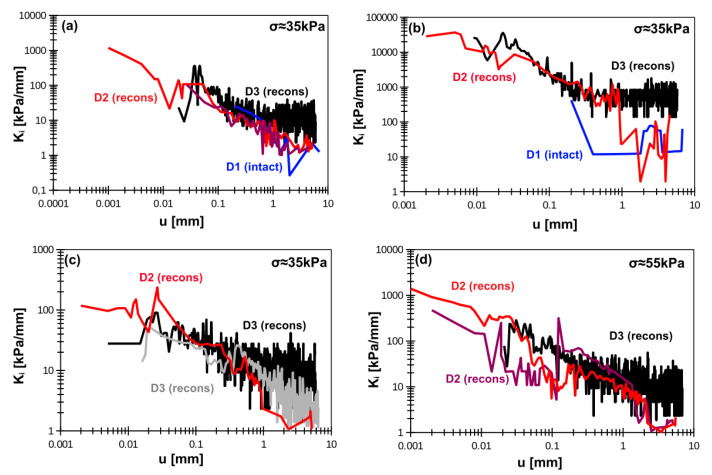
Smooth concrete–soil interface stiffness degradation for (**a**) silty loam, (**b**) organic clay, (**c**) peat, (**d**) organic silt. Note: D1, D2, D3 indicate device type; intact = intact sample, recons = reconstituted sample.

**Figure 8:**
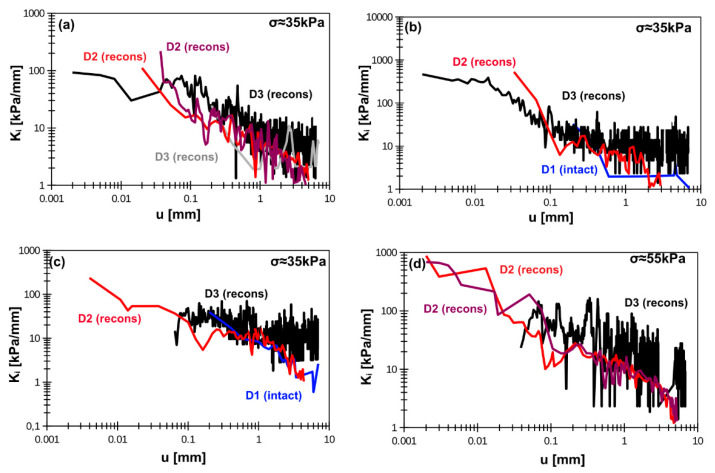
Rough concrete–soil interface stiffness degradation for (**a**) silty loam, (**b**) organic clay, (**c**) peat, (**d**) organic silt. Note: D1, D2, D3 indicate device type; intact = intact sample, recons = reconstituted sample.

The degradation of the interface stiffness (K_i_) with displacement is presented in Figure 7 and Figure 8 for smooth and rough interfaces, respectively. The reliability in stiffness measurements starts around displacement of approximately 0.01 mm. The interface stiffness almost fades away at the displacement between 0.5 and 1 mm. The initial value of K_i_ is the most important one. Here, authors delivered initial value as that corresponding to displacement equal to approximately 0.05 mm. The reason is that between 0.01 and 0.05 mm the calculated stiffness usually exhibits oscillations and establishing the proper value has to be done by engineering judgment. The oscillations are induced by different shear force increase in the same displacement intervals. Consequently, the calculated tangent stiffness can have higher or lower values.

**Figure 9:**
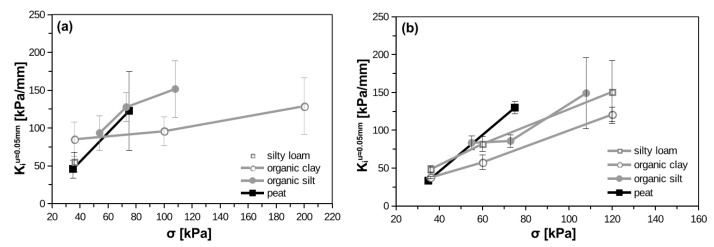
Stress dependency of interface shear stiffness: (**a**) smooth interface, (**b**) rough interface.

Interface stiffness–stress dependency is presented in Figure 9. As one can see, the application of smooth interface results in larger variability of interface stiffness. The interface stiffness increases with normal stress. The interface stiffness parameters in forward and backward shearing are summarized in Table 7 and Table 8 for smooth and rough interfaces, respectively. Authors decided that for typical engineering application and typical stress range (30 to 100 kPa) the interface stiffness for 0.05 mm can be assumed as the initial one (K_i0_). The variability of K_i_ also suggests estimation of the one, strict value regardless interface type. Consequently, the values of K_i0_ in Table 7 and Table 8 are chosen by engineering judgment. The interface stiffness in backward shearing (for the same range of displacement) is generally 2 to 4 times higher. However, large variability of that value was observed.

## 5. Interface Hyperbolic Model and Instrumented Pile Tests

### 5.1. Hyperbolic Model

Hyperbolic model captures the soil and interface non-linear behavior and stress-dependency. It also predicts in easy way the interface stiffness degradation. The classic hyperbolic model for pile–soil interface can be written as [9,37]:

(7) $$\tau =\frac{u}{\frac{1}{K_{i0}}+\frac{u}{\tau_{\max}}}$$

where: τ = shear stress on pile-soil interface; u = displacement; K_i0_ = initial interface stiffness; τ_max_ = maximum shear stresses on the interface. In present study, the τ_max_ can be assumed with Mohr-Coulomb envelopes presented in Figure 4 and Figure 5.

### 5.2. Instrumented Pile Tests

**Figure 10:**
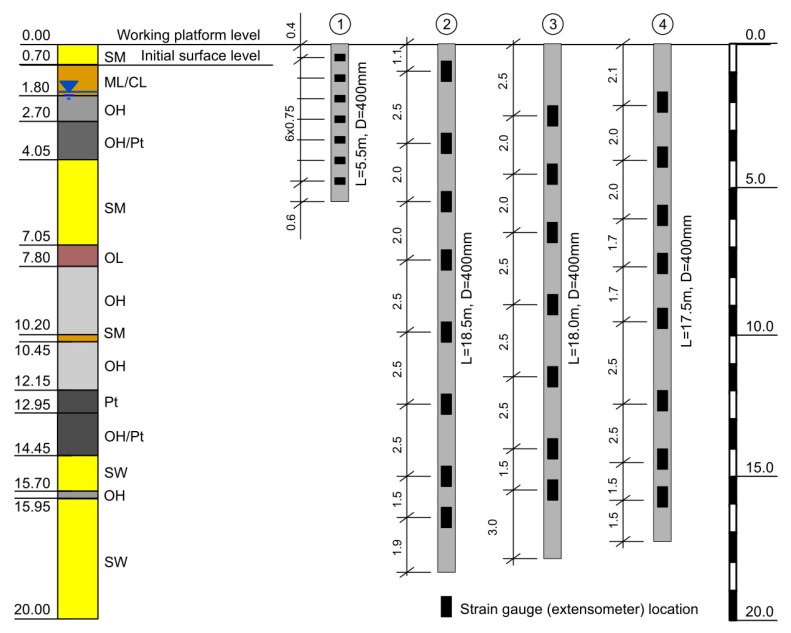
Instrumented piles schemes and corresponding soil profile on testing field.

Instrumented piles are widely used to determine pile shaft and base unit resistances and load transfer characteristics [38,39,40,41]. Instrumented pile is equipped with strain gauges located at several depths in order to measure axial strain. This allows to calculate the axial forces in each pile section under different load levels [42]. Consequently, the skin friction–displacement behavior can be obtained. In the trail fields located in the Vistula Marshlands, four instrumented controlled modulus columns (CMC) with lengths of 17.5–18.5 m (3 piles) and 5.5 m (1 pile) were tested in compression static loading tests. The piles were equipped with 7 (2 piles) or 8 (also 2 piles) vibrating wire strain gauges at different depth to measure the skin friction in each soil layer, see Figure 10. Based on these measurements, the skin friction mobilization and load transfer curves for silty loam, organic clay, peat (5.5 m length pile), and organic silt (17.5–18.5 m length piles) were determined.

### 5.3. Comparison between Hyperbolic Model and Instrumented Pile Tests

**Figure 11:**
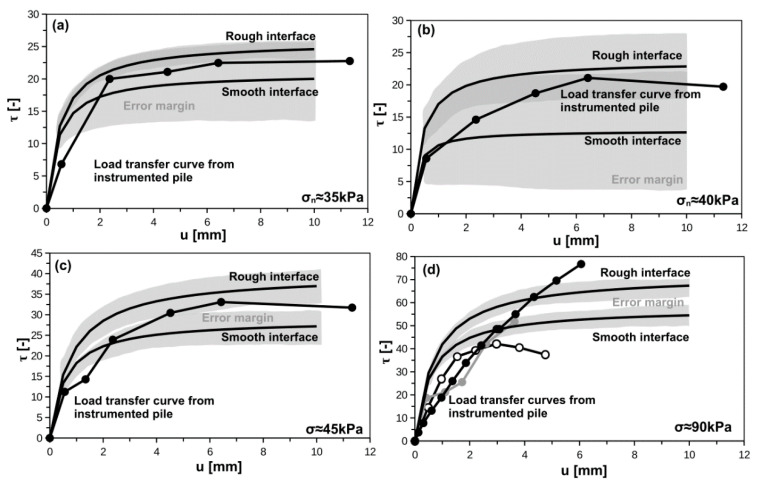
Interface hyperbolic model versus load transfer curves from instrumented pile static loading tests: (**a**) silty loam, (**b**) organic clay, (**c**), peat, (**d**) organic silt.

The comparisons between interface testing and hyperbolic interface model on the one side, and results from static loading tests of instrumented piles on the other, are presented in Figure 11. Authors shown the average values for rough and smooth interfaces and variability based on standard errors of parameters (see Table 7 and Table 8 for initial stiffness interface, and Figure 4 and Figure 5 for maximum interface shear stress). The normal stress applied in interface shear box for a given soil type corresponds to the normal stress acting on the pile shaft. The normal load acting on pile shaft is assumed to be equal to horizontal in-situ stress, which was confirmed by dilatometer soundings conducted 30 cm from the pile shaft [43].

As one can see, for silty loam, organic clay and peat, the field data are located between rough interface and smooth interface. However, the error margin is very large for organic clay, moderate for silty loam, and relatively low for peat. In terms of organic silt, the field data are characterized by large scatter and they barely fit the hyperbolic curve model. Two field results seem to fit the data from lab tests while one is significantly higher than others. It can be related to specific conditions: e.g., unique installation of the CMC, which may cause higher horizontal stress on the interface itself. The real CMC diameter in organic silt layer can be also slightly different than nominal value of 400 mm (independent construction event) what results in overestimation of the axial forces in instrumented pile and shear stresses consequently. In almost all cases the initial stiffness from instrumented piles were slightly lower than estimated form lab tests. Based on the above comparison, one can noticed that lab determination of the soil interface characteristic can be successfully used in conjunction with hyperbolic model for modeling pile-soil interface behavior.

## 6. Conclusions

This paper presents the stiffness and shear characteristics of soft soil–concrete interfaces and applicability of hyperbolic interface model to predict load transfer curves for pile shaft. Presented research provides basic shear and stiffness parameters for selected soft soils and expands knowledge about variability of interface characteristics. The presented study allows to following conclusion to be drawn:

1. Intact samples usually exhibit lower interface shear strength then reconstituted ones;
2. There is significant variability on adhesion that influences the hyperbolic interface model. For instance, organic clay–concrete interface shear strength can vary from 5 to 25 kPa (Figure 10) depending on interface type. The reasons for discrepancies can be related to the microscopic random arrangement of soil particles and small peats inserts (also located randomly within the sample);
3. Backward shearing following forward reduces interface shear strength up to 35% depending on interface and organic soil type;
4. Shear stiffness increases with normal stress and drops with displacement level. It is also characterized by significant scatter;
5. Shearing in backward following forward direction produces usually 2 or 3 times higher initial stiffness than shearing directly in forward direction;
6. Organic silts and peats can be characterized by the lowest variability of results while organic clays by the highest;
7. Shear and stiffness parameters obtained in this research fit the literature data;
8. Interface shear testing with hyperbolic model can be used to determine load transfer curves that fits the field measurements of instrumented piles during static loading test;
9. The variability and error propagation can be useful in determination of interface shear strength variability and the upper and lower bound of mobilized skin friction.

## Figures and Tables

**Table 1:** Smooth concrete interface testing dataset.

Soil Type	δ	c_a_	ϕ’	c’	δ/ϕ’	τ_f_/τ_b_	K_i_	σ_n_	u	Reference
	(°)	(kPa)	(°)	(kPa)	(-)	(-)	(kPa/mm)	(kPa)	(mm)	
Red clay	24 ^(1)^	-	-	-	-	1.13	102 ^(2)^	100	-	[22]
Red clay	10	41	16	95	0.63	-	-	-	-	[20]
Sandy clay	18.3	14	28	16.2	0.65	-	200	100	0.1	[18]
Silt	22.3–26.5	-	29–30.5	0	0.74–0.90	-	-	-	-	[25]
Silt	25.2–27.7	-	27–30	0	0.92–0.93	-	-	-	-	[19]
Silt	-	-	-	-	1.0	-	-	-	-	[16]

Note: δ = interface friction angle; c_a_ = adhesion; ϕ’ = effective angel of internal friction; c’ = effective cohesion; τ_f_/τ_b_ = forward to backward shear strength ratio; K_i_ = interface shear stiffness at σ_n_ and u; σ_n_ = normal stress (CNL); u = horizontal displacement; ^(1)^ = estimated point value from one measurement; ^(2)^ = secant stiffness.

**Table 2:** Rough concrete interface testing dataset.

Soil Type	δ	c_a_	ϕ’	c’	δ/ϕ’	τ_f_/τ_b_	K_i_	σ_n_	u	Reference
	(°)	(kPa)	(°)	(kPa)	(-)	(-)	(kPa/mm)	(kPa)	(mm)	
Peat ^(1)^	33.8	-	43	-	0.79	-	100	140	0.2	[21]
Marine Leda clay	33–35	-	30.5	0	1.11	-	312	250	0.04	[26]
Red clay	26.5–28.5 ^(2)^	-	-	-	-	1.36–1.54	105 ^(3)^	100	-	[22]
Red clay	10–13	41–104	16	95	0.63–0.81	-	-	-	-	[20]
Illite clay	25	7	26	9	0.96	-	200	50	0.1	[27]
Clay	26.5–40 ^(4)^	7	34	50	0.78–1.17	-	75	30–60	0.2	[17]
Silty clay	31.4–34.5	4.6–10.4	33.1	18.5	0.95–1.04	-	20	50	1	[24]
Sandy clay	21	15	28	16.2	0.75	-	670	100	0.06	[18]
Silt	30	-	29–30.5	0	0.98–1.02	-	-	-	-	[25]

Note: δ = interface friction angle; c_a_ = adhesion; ϕ’ = effective angel of internal friction; c’ = effective cohesion; τ_f_/τ_b_ = forward to backward shear strength ratio; K_i_ = interface shear stiffness at σ_n_ and u; σ_n_ = normal stress (CNL); u = horizontal displacement; ^(1)^ = 10% sand in peat sample; ^(2)^ = estimated point value from one measurement; ^(3)^ = secant stiffness; ^(4)^ = range depends on external (higher values) or internal (lower values) measurement.

**Table 3:** Geotechnical parameters of soils used in interface testing.

No	Soil	Type	Soil Properties							Intact	Reconstituted
			LOI	w	ρ	k	E^oed^	ϕ’_max_	c’		
			(%)	(%)	(g/cm^3^)	(m/s)	(MPa)	(°)	(kPa)		
1	Silty loam	NC	5.1	30.6	1.74	2.50e–7 ^(1)^	1.6–2.3	30.9 ^(3)^	0	˅	˅
2	Organic clay	NC	11.9	68.0	1.47	2.46e–7	0.9–2.3	23.1 ^(2)^–26.4 ^(3)^	0	˅	˅
3	Organic silt	NC	7.0	49.3	1.63	4.84e–8	4.2–5.2	31.3 ^(2)^–36.6 ^(3)^	0	x	˅
4	Peat	NC	78.5	270.1	1.18	2.73e–6	1.2–1.8	55.6 ^(2)^	0	˅	˅

Note: NC = normally consolidated; LOI = loss on ignition; w = water content; ρ = soil density; k = coefficient of permeability; E^oed^ = oedometric modulus; ϕ’_max_ = maximum effective angle of internal friction; c’ = effective cohesion; intact = intact sample; reconstituted = reconstituted sample; ˅ = sample available; x = sample not available; ^(1)^ = value estimated from oedometer test; ^(2)^ = triaxial consolidated drained or undrained test, ^(3)^ = direct shear box (consolidated, drained test).

**Table 4:** Interface shear strength testing program.

Soil	Number of Tests ^(1)^		
	Repeatability	Fatigue in Backward Shearing	Stiffness Degradation
Silty loam	4 (4)	3 (5)	4 (4)
Organic clay	3 (3)	4 (4)	3 (3)
Organic silt	3 (3)	5 (3)	3 (3)
Peat	4 (3)	5 (4)	3 (3)

^(1)^ number indicate smooth interface and number in bracket denotes rough interface.

**Table 5:** Friction characteristics of smooth concrete–soft soil interfaces.

Soil	Interface Shear Strength Parameters				
	δ_f_	tan(δ_f_) ± S_tan(δf)_	c_a_ ± S_ca_	τ_f_/τ_b_	δ_f_/ϕ’_max_
	(°)	(-)	(kPa)	(-)	(-)
Silty loam	24.1	0.448 ± 0.024	5.1 ± 3.7	1.09 ÷ 1.30	0.78
Organic clay	14.4	0.256 ± 0.026	12.6 ± 5.1	0.91 ÷ 1.43	0.62
Organic silt	31.3	0.607 ± 0.010	3.0 ± 2.9	0.89 ÷ 1.09	1.00
Peat	26.4	0.490 ± 0.016	6.7 ± 2.4	1.00 ÷ 1.25	0.47

Note: δ_f_ = angle of interface friction in forward shearing; S_tan(δf)_ = standard error of tan(δ_f_); c_a_ = adhesion; S_ca_ = standard error of c_a_; τ_f_/τ_b_ = forward to backward shear strength ratio; ϕ’_max_ = maximum angle of internal friction.

**Table 6:** Friction characteristics of rough concrete–soft soil interfaces.

Soil	Interface Shear Strength Parameters				
	δ_f_	tan(δ_f_) ± S_tan(δf)_	c_a_ ± S_ca_	τ_f_/τ_b_	δ_f_/ϕ’_max_
	(°)	(-)	(kPa)	(-)	(-)
Silty loam	30.6	0.591 ± 0.029	5.1 ± 2.3	0.92 ÷ 1.15	0.99
Organic clay	21.8	0.400 ± 0.053	7.7 ± 4.4	1.13 ÷ 1.31	0.94
Organic silt	33.8	0.672 ± 0.028	11.7 ± 2.4	0.92 ÷ 0.98	1.08
Peat	29.2	0.559 ± 0.016	11.9 ± 2.6	0.95 ÷ 1.28	0.52

Note: δ_f_ = angle of interface friction in forward shearing; S_tan(δf)_ = standard error of tan(δ_f_); c_a_ = adhesion; S_ca_ = standard error of c_a_; τ_f_/τ_b_ = forward to backward shear strength ratio; ϕ’_max_ = maximum angle of internal friction.

**Table 7:** Interface stiffness characteristics for smooth concrete–soil interfaces.

Soil	Interface Stiffness	
	K_i0_ ≈ K_if0_ (u ≈ 0.05mm, σ ≈ 50kPa)	K_ib0_/K_if0_
	(kPa/mm)	(-)
Silty loam	50 ± 10	2.8 ÷ 3.0
Organic clay	60 ± 20	2.7 ÷ 4.0
Organic silt	100 ± 20	1.2 ÷ 2.3
Peat	50 ± 20	1.3 ÷ 1.8

Note: K_i0_ = initial interface stiffness; K_if0_ = interface stiffness in forward shearing; K_ib0_ = interface stiffness in backward shearing.

**Table 8:** Interface stiffness characteristics for rough concrete–soil interfaces.

Soil	Interface Stiffness	
	K_i0_≈K_if0_ (u ≈ 0.05mm, σ ≈ 50kPa)	K_ib0_/K_if0_
	(kPa/mm)	(-)
Silty loam	50 ± 10	2.2 ÷ 2.6
Organic clay	60 ± 20	2.9 ÷ 4.2
Organic silt	100 ± 20	2.4 ÷ 4.0
Peat	50 ± 20	1.4 ÷ 2.3

Note: K_i0_ = initial interface stiffness; K_if0_ = interface stiffness in forward shearing; K_ib0_ = interface stiffness in backward shearing.

## Data Availability

The data presented in this study are available on request from the corresponding author.
